# Exploring Caregivers Lived Experiences in Accessing Comprehensive Cleft Care at Jimma University Medical Center: A Phenomenological Study in Southwest Ethiopia

**DOI:** 10.3390/children13101372

**Published:** 2026-10-10

**Authors:** Abiy Semunigus Dejene, Allyn Auslander, Abraham Tamirat Gizaw, Fira Abamecha Ababulgu, Elias Ali Yesuf

**Affiliations:** 1 Department of Health, Behavior, and Society, Faculty of Public Health, Institute of Health, Jimma University, Jimma P.O. Box 378, Ethiopia; 2 Operation Smile, Virginia Beach, VA 23453, USA; 3 Department of Health Policy and Management, Faculty of Public Health, Institute of Health, Jimma University, Jimma P.O. Box 378, Ethiopia

**Keywords:** comprehensive cleft care, orofacial clefts, lived experience, phenomenological study

## Abstract

**Highlights:**

**What are the main findings?**

- Caregivers perceived comprehensive cleft care as transformative, with reported improvements across clinical, nutritional, psychosocial, and social domains. Participants described perceived improvements in feeding, health, physical appearance, caregiver confidence, and social reintegration following engagement with coordinated nutritional, surgical, speech, dental, and psychosocial services.
- Caregivers reported that access and continuity of care were constrained by interconnected social, economic, cultural, and health system barriers: Limited awareness, stigma and misconceptions, delayed referrals, geographic distance, transportation costs, and financial hardship were reported as factors that delayed treatment and disrupted continuity of care.

**What are the implications of the main findings?**

- The findings underscore the importance of strengthening integrated, comprehensive cleft care services within public health facilities, including surgery, nutritional rehabilitation, psychosocial support, caregiver education, and coordinated follow-up care to better support children with orofacial clefts and their families.
- Improving equitable access to comprehensive cleft care requires addressing transportation and financial barriers, strengthening referral pathways and community awareness, and reducing stigma and misconceptions.

**Abstract:**

**Background:** Orofacial clefts are one of the most common congenital anomalies worldwide. Comprehensive cleft care interventions aim to address the complex surgical, nutritional, dental, speech, audiology, and psychosocial needs of patients with clefts. While studies from other countries have identified various implementation barriers and facilitators, evidence from Ethiopia remains limited, particularly regarding caregivers’ experiences accessing comprehensive cleft care. This study explored caregivers’ lived experiences of accessing comprehensive cleft care at Jimma University Medical Center in southwestern Ethiopia. **Methods:** We conducted a phenomenological study at the Comprehensive Cleft Care Center of Jimma University Medical Center. Participants were purposively selected from caregivers of children with orofacial clefts who had received at least two components of comprehensive cleft care. Data were collected through in-depth semi-structured interviews. Data collection continued until thematic saturation was achieved. Audio recordings were transcribed verbatim, translated into English, and managed in ATLAS.ti version 7.5.18. Data were analyzed inductively using Colaizzi’s seven-step descriptive phenomenological method. Trustworthiness was enhanced through member checking, reflexive bracketing, and systematic procedures. **Results:** Twelve caregivers of children with orofacial clefts participated in the study. The analysis generated eight overarching themes. Participants reported limited initial awareness of cleft conditions, cultural misconceptions, stigma, emotional distress, and delayed care-seeking. Key barriers included poor referral linkages, transportation difficulties, financial constraints, and geographic inaccessibility. Participants reported that engagement with comprehensive cleft care was associated with increased knowledge, caregiving confidence and skills, and trust in healthcare providers. They also described perceived improvements in feeding, nutritional status, speech development, physical appearance, and social integration, alongside reduced stigma and increased caregiver advocacy and community awareness of cleft conditions. **Conclusions:** Caregivers described a transition from limited awareness of orofacial clefts, emotional distress, stigma, and challenges accessing care toward greater knowledge, confidence, trust in healthcare providers, and perceived improvements in their children’s health, functional abilities, and social participation. Persistent barriers related to poverty, transportation difficulties, referral delays, and limited community awareness remained. These findings can inform policy and program planning to strengthen comprehensive cleft care and address inequities in access to care for children with orofacial clefts and their families in similar settings in Ethiopia.

## 1. Introduction

Globally, orofacial clefts (OFCs) are among the most common congenital anomalies [1,2]. They arise from a complex interplay of genetic and environmental factors during early embryonic development [2]. Global estimates suggest OFCs affect about 1 in 700 births worldwide, with more than 200,000 new cases each year [3,4].

The burden of OFCs is unevenly distributed across populations, with many low- and middle-income countries (LMICs) facing challenges related to limited healthcare resources, specialized expertise, and access to appropriate services as part of broader efforts to strengthen oral health systems and achieve universal health coverage [4,5,6,7]. In Ethiopia, the estimated prevalence of OFCs is approximately 1.4 per 1000 live births, corresponding to more than 5000 new cases annually [4,8,9]. Beyond facial appearance, OFCs may affect important aspects of health and functioning, including feeding, nutrition, speech, hearing, and dental health [7,10,11,12]. Children with OFCs may experience feeding difficulties that can contribute to inadequate nutritional intake and poor growth, while stigma and limited community awareness may further affect the social participation and well-being of children and their families [10,13,14].

Comprehensive cleft care (CCC) is increasingly recognized as an integrated approach to cleft care that emphasizes multidisciplinary, longitudinal, safe, and sustainable management across the continuum of care delivered through coordinated cleft teams [15,16]. It encompasses a range of services, including nutritional support, dental care, psychosocial counseling, audiological management, speech therapy, surgical treatment, and long-term follow-up, reflecting the broad functional outcomes targeted by comprehensive cleft-care programs [11,16,17,18]. Historically, cleft care in Ethiopia has often relied on intermittent surgical missions, with limited continuity of multidisciplinary care and follow-up. More recently, institutions such as Jimma University Medical Center (JUMC) have increasingly adopted hospital-based multidisciplinary cleft-care models consistent with international standards for comprehensive cleft care [16,19].

Despite the development of hospital-based cleft-care programs, access to specialized services remains challenging in Ethiopia. Specialized surgical services in resource-constrained settings can strengthen local capacity while also highlighting the importance of specialist expertise, infrastructure, surgical workforce capacity, and sustained institutional support [20,21]. These considerations are relevant to the continued development and decentralization of comprehensive cleft care in Ethiopia. Reported barriers include long-distance travel, financial constraints, sociocultural factors that may delay care-seeking, and limited access to specialized services [5,12,13,22,23]. Nutritional status can also affect access to surgical treatment, with children who are not adequately nourished sometimes requiring nutritional rehabilitation before surgery [24,25].

Despite growing evidence on the epidemiology, clinical characteristics, and treatment outcomes of orofacial clefts in Ethiopia [4,9,17,26], little is known about how caregivers experience and navigate comprehensive cleft care over time. Existing studies have primarily focused on disease burden, treatment outcomes, and access to surgical services, while qualitative evidence on caregiver experiences has largely emerged from other settings, including Ghana, Rwanda, Nigeria, India, Europe, and other low- and middle-income countries [22,27,28,29,30,31,32,33,34]. As comprehensive cleft care models expand in Ethiopia, understanding caregivers’ perspectives across the continuum of care is essential for informing patient- and family-centered service delivery.

This study therefore used a qualitative descriptive phenomenological approach to explore the lived experiences of caregivers of children with OFCs receiving care through the JUMC Comprehensive Care program. By examining caregivers’ experiences, perceptions, and challenges throughout the care pathway, the study seeks to provide context-specific evidence on the delivery and experience of longitudinal multidisciplinary cleft care in Ethiopia.

## 2. Materials and Methods

### 2.1. Study Design and Setting

Jimma University Medical Center (JUMC) is one of the largest teaching and referral hospitals in southwestern Ethiopia. The hospital operates under Jimma University and is in Jimma, approximately 353 km from the capital city, Addis Ababa. One specialized healthcare area that has gained significant attention at JUMC is the treatment of cleft lip and cleft palate, congenital conditions that can affect feeding, speech, hearing, dental development, psychosocial well-being, and quality of life. These complex conditions require coordinated, multidisciplinary healthcare services.

In 2005, JUMC partnered with Operation Smile, the world’s largest cleft care organization, which provides free comprehensive cleft care services worldwide. This collaboration has been a major milestone in improving access to cleft treatment in southwestern Ethiopia. This partnership reached a major milestone in 2022 with the establishment of a Comprehensive Cleft Care Center at Jimma University Medical Center and Operation Smile.

This center marked a shift from short-term surgical outreach programs to a sustainable, patient-centered care model. It includes multidisciplinary services such as surgical care, nutritional assessment and rehabilitation, dental services, speech and language support, psychosocial support, and longitudinal follow-up. These services address the changing clinical, functional, nutritional, and psychosocial needs of children with cleft conditions across the care continuum. The study was conducted from 27 May 2026 to 30 July 2026.

### 2.2. Study Design

The study used a descriptive phenomenological design to examine caregivers’ lived experiences of accessing comprehensive cleft care at Jimma University Medical Center.

### 2.3. Source Population

The source population was caregivers of orofacial cleft patients who attended comprehensive cleft care centers at Jimma University Medical Center.

### 2.4. Study Population

The study population included caregivers of orofacial cleft patients who came to JUMC to access comprehensive cleft care during the study period and attended more than two types of comprehensive cleft care interventions.

### 2.5. Sample Size Determination

Data collection continued until thematic saturation, when subsequent interviews yielded no new information or themes. During the interview process, we reviewed data concurrently with collection and conducted preliminary analysis. After the tenth interview, we identified no substantially new meanings, concepts, or experiences related to caregivers’ lived experiences of comprehensive cleft care. To confirm saturation, the researcher conducted two additional interviews.

The interviews confirmed the existing themes and did not generate any new codes, categories, or experiential insights. Given the lack of new information and the redundancy of meanings already identified, the researcher decided that information saturation had been reached after the twelve interviews. Therefore, the researcher terminated participant recruitment and data collection.

### 2.6. Sampling Techniques and Procedures

A purposive sampling technique was utilized. Participants were purposively selected from caregivers who had attended at least two comprehensive cleft care interventions, including surgery, nutrition, speech therapy, psychosocial care, and dentistry. This criterion was intentionally applied to ensure that participants had sufficient experience with multiple encounters and components of the care pathway to provide information-rich accounts of accessing, navigating, and receiving comprehensive cleft care. Caregivers whose children had attended only one care encounter or had not accessed the JUMC Comprehensive Cleft Care program were ineligible to participate.

Data were collected through in-depth semi-structured interviews, and sampling continued until data saturation was reached, when no new themes or insights emerged. Data collection and analysis were conducted iteratively. The primary researcher reviewed interview data as interviews progressed, comparing emerging codes and categories with those identified in previous interviews. Thematic sufficiency was considered when subsequent interviews no longer contributed substantially new experiential dimensions relevant to the study objectives.

After ten interviews, the emerging themes and subthemes were sufficiently developed, with subsequent interviews primarily reinforcing previously identified patterns. Two additional interviews were conducted to further assess the adequacy and depth of the emerging findings. Following these interviews, no new themes or meaningful experiential dimensions emerged, and data collection was concluded after 12 interviews.

### 2.7. Data Collection and Tool

Semi-structured interviews explored caregivers’ experiences of service access, care delivery, and care coordination. The primary researcher, who has postgraduate training in public health, conducted the interviews. A trained qualitative researcher fluent in both Afaan Oromo and Amharic participated as a second data collector. Before each interview, the researcher built rapport with participants and explained his academic role, the study’s purpose, and the interview objectives.

The researcher had no direct clinical or supervisory relationship with the participants that could influence their participation or responses. Participants were informed that participation was voluntary, that their responses would be kept confidential, and that participation would not affect their access to care or services.

Interviews were conducted individually in a private room at JUMC, with no nonparticipants present. The researchers documented field notes to capture contextual information, observations, and reflections relevant to interpreting the data.

The interview guide included 42 questions, and interviews remained open-ended and participant-centered, allowing participants to describe their experiences in their own words. Each in-depth interview lasted approximately 40 to 90 min and was conducted in the participant’s preferred language (Afaan Oromo or Amharic). With participants’ informed consent, we audio-recorded all interviews. No repeat interviews were conducted.

### 2.8. Data Analysis Procedure

Interviews were conducted in either Afaan Oromo or Amharic, based on participants’ language preference, and were audio-recorded with their consent. Each interview was transcribed verbatim in the original language before translation into English. The principal investigator and one qualitative data collector, fluent in both Afaan Oromo and Amharic and experienced in qualitative research, performed the translation.

The English translations were reviewed against the original-language transcripts to assess consistency and preserve the intended meaning of participants’ accounts. The principal investigator and data collector resolved any discrepancies or ambiguities identified during the review by consensus. Participant quotations presented in the manuscript represent translated versions of the original statements. We made minor grammatical and editorial adjustments to improve readability in English while preserving the original meaning.

The principal investigator and qualitative data collector read each transcript alongside the corresponding audio file to verify the accuracy and completeness of the transcriptions. Data were managed using ATLAS.ti version 7.5.18 to facilitate data organization, retrieval, and coding. We analyzed the data inductively using Colaizzi’s seven-step descriptive phenomenological method [35]. We read all transcripts several times to gain a general understanding of caregivers’ experiences and identified and extracted significant statements relevant to the phenomenon under study.

We then formulated meanings from these significant statements while remaining faithful to the participants’ accounts. We grouped similar meanings into clusters of themes and subthemes that reflect common patterns across participants. The resulting theme clusters were integrated into an exhaustive description of the phenomenon, from which the fundamental structure representing the essence of caregivers’ lived experiences was developed.

To formulate meaning and organize it into clusters, after extracting significant statements (Step 2 of Colaizzi’s method), the researcher proceeded to Step 3, in which the underlying meaning of each significant statement was carefully interpreted while remaining faithful to the participants’ original descriptions. These formulated meanings represented the essential meanings embedded within the caregivers’ narratives of their lived experiences.

**Figure 1:**
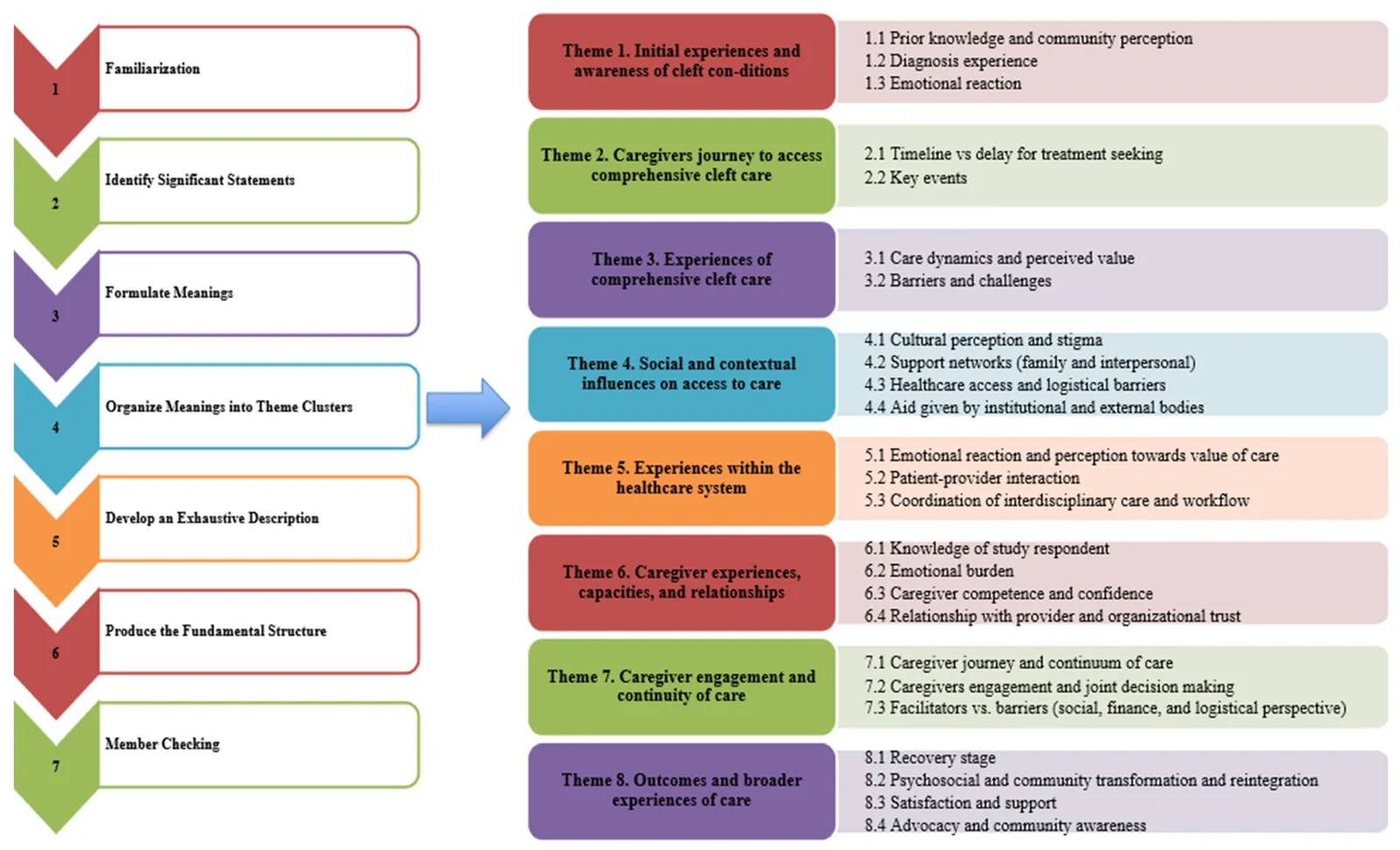
Steps of Colaizzi’s phenomenological analysis and the formation of the main themes of caregivers’ lived experiences accessing comprehensive cleft care at Jimma University Medical Center in Southwest Ethiopia, 2026.

In Step 4, we grouped meanings with similar content into theme clusters based on conceptual similarities and shared meaning. We continually compared these theme clusters with the original interview transcripts to determine whether each theme accurately reflected participants’ accounts and was grounded in the data. Through this iterative analytical process, we identified approximately twenty-one subthemes and organized them into eight overarching themes that represent the essential structure of caregivers’ lived experiences of accessing and receiving comprehensive cleft care at Jimma University Medical Center. Detailed steps of Colaizzi’s phenomenological analysis and the formation of the main themes found (Figure 1).

### 2.9. Trustworthiness

The team established trustworthiness using the criteria proposed by Lincoln and Guba [36]. To enhance credibility, the analysis followed systematic procedures, including verbatim transcription, repeated reading of the interview transcripts, careful review of the original interview recordings, and application of Colaizzi’s seven-step descriptive phenomenological method [35]. Following the seventh step of Colaizzi’s method, the researcher presented a summary of the findings to three randomly selected participants from the 12 participants for member checking. Participants assessed whether the identified themes accurately reflected their lived experiences and whether any important aspects had been misunderstood or omitted. Participants confirmed that the findings broadly reflected their experiences and provided minor clarifications regarding the wording and interpretation of some findings.

The primary researcher led coding, meaning-making, and the development of themes and subthemes. An experienced qualitative researcher independently reviewed two of the twelve transcripts as an additional analytic quality check. The review examined whether the coding and emerging interpretations were grounded in participants’ narratives and whether alternative interpretations were warranted. The team discussed and resolved any differences in interpretation by returning to the relevant transcripts and participants’ original accounts. Together, these procedures provided multiple checks on the credibility, dependability, and confirmability of the findings.

Trustworthiness was enhanced through reflexive practices throughout data collection and analysis. The primary researcher consciously acknowledged his prior professional knowledge and familiarity with the study context, developed through five years of cleft care experience. The primary researcher used open-ended, non-leading questions to allow participants to describe their experiences in their own words. During analysis, the primary researcher repeatedly checked interpretations against participants’ original narratives, including accounts that differed from emerging interpretations.

Themes and subthemes emerged inductively from the data rather than being imposed by a predetermined conceptual framework. This process helped maintain a close connection between the participants’ accounts and the final interpretations. Negative case analysis examined data that did not support or contradict the emerging themes, adding nuance to the interpretation and providing a complete picture of participants’ experiences. Transferability was achieved by detailing the study setting, participants, data collection procedures, and analytical process, allowing readers to judge the findings’ applicability to comparable settings.

### 2.10. Ethical Consideration

The Jimma University Institutional Review Board granted ethical approval on 25 May 2026 (Ref No: JUIH/IRB/0587/2026). The study team obtained verbal and written informed consent from study participants. Participation was voluntary, and participants could withdraw at any time. The team ensured confidentiality through coded identifiers, anonymized transcripts, and secure storage of audio recordings and field notes (Supplementary File S1).

## 3. Results

Twelve caregivers of children with orofacial clefts participated in the study. Their accounts described experiences from initial recognition of the cleft condition through the treatment-seeking journey, engagement with comprehensive cleft care, and subsequent changes in caregiving, functioning, and social participation. The analysis yielded eight overarching themes and twenty-one subthemes.

### 3.1. Participants’ Demographic Profile

This study included twelve in-depth interview respondents: one male and eleven female participants. This included one father, one grandmother, and ten mothers. All study participants were receiving care at Jimma University Medical Center for their children with cleft conditions. Participants ranged in age from 22 to 38 years, with a mean age of 27.5 years. All study participants reside in the Oromia and Southwest regions of Ethiopia (Table 1).

### 3.2. Qualitative Analysis of Lived Experiences of Caregivers in Accessing Comprehensive Cleft Care at Jimma University Medical Center

The study identified eight themes reflecting caregivers’ lived experiences in accessing comprehensive cleft care at JUMC. These include initial experiences and awareness of cleft conditions, caregivers’ journey to access comprehensive cleft care, experience of receiving comprehensive cleft care, social and contextual influences on access to care, experiences within the healthcare system, caregiver adaptation and capacity, caregiver engagement and continuity of care, and perceived changes and broader experiences of care. Detailed classification of themes and sub-themes is provided in Table 2.

Main Theme 1: Initial Experiences and Awareness of Cleft Conditions

Sub-theme 1.1: Prior Knowledge and Community Perceptions

Most caregivers reported having little or no prior knowledge of orofacial cleft before their child was born. Several had never seen or heard about cleft conditions and therefore did not know that they could be treated. Community beliefs and explanations strongly influenced their initial understanding. Some participants indicated beliefs that a cleft could result from a pregnant woman looking at, laughing at, or interacting with a person with a cleft. These beliefs contributed to confusion and fear following the birth of their children. “I had never seen such a child before. It was the first time I saw a child in this condition when I gave birth. The neighbors said that I had laughed at someone with a cleft and that was why my child was born like this” (P-1).

Sub-theme 1.2: Diagnosis Experience

Interviewees described the diagnosis as unexpected and distressing. Most children were diagnosed shortly after birth, particularly when a cleft lip was visibly apparent. In contrast, some caregivers reported recognizing a cleft palate later, often because of feeding difficulties, poor weight gain, or clinical assessment. Few caregivers reported receiving information about the condition during pregnancy through antenatal ultrasound. The unexpected diagnosis often leaves caregivers uncertain about the child’s future and about where to seek appropriate care. One caregiver described the immediate uncertainty following the birth of her child: “I was shocked and worried because I had never seen this condition before. I did not know what would happen to my child in the future” (P-2).

Sub-theme 1.3: Initial Emotional Response

The diagnosis generated substantial emotional distress among caregivers. Participants described feelings of shock, heartbreak, anxiety, loneliness, stress, and persistent crying. Some caregivers reported difficulty accepting their child’s condition, while others described withdrawal from social interaction and loss of appetite. The emotional burden was sometimes intensified by uncertainty about the child’s future, stigma, and limited understanding of the condition. In a few accounts, caregivers described extremely severe emotional distress, including thoughts of death. “I was very anxious, worried, and stressed. I cried a lot. My husband could not accept the child and did not eat for two days. I prayed to God to take the child’s life or even my own” (P-2).

Main Theme 2: Caregivers’ Journey to Access Comprehensive Cleft Care

Sub-theme 2.1: Treatment-Seeking Journey and Delays

Respondents noted the pathway to comprehensive cleft care as complex and non-linear. Families often moved through health posts, health centers, and referral hospitals before reaching JUMC. Delays were associated with limited knowledge about cleft treatment, inadequate referral information, geographical distance, transportation difficulties, and lack of money. Some caregivers initially remained at home because they did not know where to seek treatment or could not afford transportation.

The timing of treatment-seeking varied considerably. Some caregivers sought care soon after birth, whereas others waited weeks or months before reaching an appropriate facility. Feeding difficulties, social isolation, and uncertainty about the condition often continued during this period. Surgical treatment could also be delayed until the child had adequate nutritional status and was medically fit for surgery. One caregiver described remaining at home for almost a month because of financial and access barriers: “We stayed at home for 28 days. The child was crying, and I was also crying because I did not know what to do. I thought the child might die” (P-3).

Sub-theme 2.2: Key Events Facilitating or Hindering Access

The journey to JUMC included both facilitating and hindering experiences. Caregivers reflected that referrals, contact with healthcare workers, community information, and assistance from Operation Smile and other supporters were important turning points. Once families reached the CCC, coordination between services, nutritional support, psychosocial assistance, and clear communication helped them continue with care.

Conversely, delayed or inappropriate referrals, prolonged waiting, transportation problems, and lack of financial resources discouraged or delayed treatment-seeking. For some caregivers, reaching a knowledgeable healthcare professional or connecting with the CCC marked a major turning point in their understanding of the condition. “When I entered the hospital, I met the Operation Smile volunteer. They accepted us and told me that the condition could be treated and that I should not worry” (P-1).

Main Theme 3: Experiences of Receiving Comprehensive Cleft Care

Sub-theme 3.1: Care Dynamics and Perceived Value of Comprehensive Care

Participants indicated comprehensive cleft care as extending beyond surgery to include nutrition, psychosocial support, speech and dental services, follow-up, and practical assistance. Participants valued the coordinated nature of these services and the continuity of support across different stages of care. Flexible appointment arrangements, clear communication, and supportive healthcare providers helped caregivers understand the care pathway and remain engaged with treatment.

Caregivers also described gaining confidence as they became familiar with the services and received guidance tailored to their children’s needs. Caregivers particularly valued nutritional support because they felt it helped their children gain strength and prepare for surgery. However, despite the availability of free or subsidized services, transportation and other costs associated with repeated visits remained important challenges. “They gave us appointments that were convenient for us and explained clearly when and where we should come. The child gained weight and became happier. The surgery was provided free of charge, and they also supported us with transportation costs” (P-3).

Main Theme 4: Social and Contextual Influences on Access to Care

Sub-theme 4.1: Cultural Perceptions and Stigma

Cultural beliefs and social stigma strongly influenced caregivers’ experiences. Participants described beliefs that clefts were caused by curses, supernatural forces, religious wrongdoing, or mistakes made by the mother during pregnancy. Some children were given derogatory labels, including “Onoka,” and caregivers described gossip, insults, social exclusion, and negative community attitudes.

These perceptions contributed to shame and reluctance to take children into public places. Some caregivers avoided social gatherings because they feared judgment or believed that others considered the child contagious. Participants also described changes in community responses following treatment, particularly when the child’s appearance or functioning improved. However, these accounts represented caregivers’ perceptions of social change rather than objectively measured changes in stigma. “People insulted us and said that the child was born because of a sin committed by the family. They called the child ‘Onoka’ and talked about us in the community” (P-1).

Sub-theme 4.2: Family and Social Support Networks

Family and social relationships shaped caregivers’ ability to cope with their children’s condition and continue accessing care. Some caregivers received practical and emotional support from spouses, relatives, neighbors, or siblings, including help with household responsibilities, transportation, childcare, and caregiving. Others described limited support, family conflict, blame, or abandonment, which increased their emotional and practical burden. In some cases, mothers carried most of the responsibility for caring for the child and managing household demands while seeking treatment.

These experiences illustrate the contrasting roles that family and social networks could play in supporting or complicating caregivers’ journeys through comprehensive cleft care. “My brother helped me by taking care of the farming activities while I was away with my child for treatment. Without his support, it would have been difficult for me to manage everything at home and continue bringing my child for care” (P-3).

Sub-theme 4.3: Healthcare Access and Logistical Barriers

Transportation and financial constraints were among the most persistent barriers to accessing care. Families traveled long distances, sometimes using multiple forms of transportation or walking substantial distances. The cost of transport, food, and accommodation accumulated across repeated visits and could require families to borrow money, sell livestock or agricultural products, or use household savings.

Some caregivers also reported environmental and contextual difficulties, including fuel shortages, interruptions in public transportation, security restrictions, illness, and poor road access. These barriers sometimes resulted in missed or delayed appointments and added stress to families already experiencing financial hardship. “Transportation was the greatest challenge. We had no stable income and had to find money each time we came. It was very difficult to continue coming for follow-up” (P-12).

Sub-theme 4.4: Institutional and External Support

Respondents noted institutional and external support as an important facilitator of continued access to comprehensive cleft care. Support included free or subsidized surgery, nutritional rehabilitation, specialized feeding materials, psychosocial support, transportation assistance, and accommodation. These forms of assistance were especially important for families with limited financial resources and helped reduce costs associated with repeated visits and treatment. Nutritional assistance was especially valued by caregivers of children who had severe feeding difficulties or were underweight. Participants described how practical support, combined with counseling and clinical care, helped them manage their children’s needs and continue with treatment. “My baby was severely underweight. They gave us free formula and a squeeze bottle and taught us how to feed the baby. Without this support, the baby would have starved. It also relieved us from the financial burden” (P-12).

Main Theme 5: Experiences Within the Healthcare System

Sub-theme 5.1: Patient–Provider Relationships and Trust

Respectful and compassionate interactions with healthcare providers were important to caregivers’ experiences of care. Participants described providers who listened to their concerns, explained treatment procedures, responded to questions, and offered reassurance. These interactions contributed to caregivers’ perceptions of being respected and valued. Trust developed over repeated encounters as caregivers became more familiar with providers and watched their children’s care progress.

Some participants contrasted their experiences at JUMC with previous experiences at other health facilities where they felt their concerns were not adequately addressed. “The staff listened to our worries and treated us with respect. They reassured us and told us what we needed to do. This made me trust them and continue with the follow-up” (P-8).

Sub-theme 5.2: Interdisciplinary Care Coordination

Caregivers valued the involvement of multiple healthcare professionals across different stages of care. They described nutrition, surgery, speech, dental, psychosocial, and follow-up services as interconnected parts of the care pathway. Participants reported that coordination between services reduced uncertainty and helped them understand the care sequence. Nutritional assessment and rehabilitation were particularly important before surgery for children who were not medically or nutritionally ready. Caregivers also described becoming more familiar with the hospital environment and service locations over repeated visits, which made subsequent navigation easier. “They started with nutrition, and after the child gained weight and became healthier, the surgery was done. The services were connected, and everything was coordinated smoothly” (P-2).

Main Theme 6: Caregiver Adaptation and Capacity

Sub-theme 6.1: Changes in Knowledge and Understanding

Participants reported changes in their understanding of cleft conditions over time. Initial explanations based on curses, supernatural causes, or community myths gradually gave way to information from healthcare providers. Counseling and health education helped caregivers understand that clefts were treatable and that care could involve several stages and different healthcare professionals.

Participants described becoming more knowledgeable about treatment requirements, follow-up, feeding, hygiene, and the importance of completing different components of care. Increased understanding also influenced how some caregivers communicated with other family members and community members about cleft conditions. “Before coming here, I knew nothing. People in our village told us that it was a curse and could not be treated. After they educated me, I understood that there was treatment and that the child could improve” (P-6).

Sub-theme 6.2: Emotional Adaptation

Caregivers’ emotional experiences changed over the course of their engagement with care. Initial despair, fear, shame, and social withdrawal were followed by greater hope and emotional acceptance for many participants. Seeing their children receive treatment and observing perceived changes in their condition were described as important sources of encouragement. Support from healthcare providers, other caregivers, family members, and faith also contributed to emotional adaptation. Some caregivers reported becoming more confident in discussing their children’s condition and less fearful of community reactions. “My heart was broken. People were talking about us, so I used to hide and cry. When I saw the children receiving surgery and the changes they were making, I started to have hope and peace” (P-5).

Sub-theme 6.3: Caregiver Competence and Confidence

Feeding was a major challenge for caregivers of children with cleft conditions. Participants described difficulties with breastfeeding, milk regurgitation through the nose, choking, coughing, and uncertainty about appropriate feeding techniques. Nutritional counseling and practical demonstrations helped caregivers learn alternative feeding positions, techniques, and practices.

Participants reported increased confidence in feeding their children and managing nutritional needs after receiving education and practical support. Some perceived subsequent improvements in feeding and growth, although these accounts reflected caregiver perceptions rather than objective nutritional measurements. “I was afraid to feed the child because milk came through the nose and the child would choke. After the training, I learned how to hold and feed the child, and I became more confident” (P-7).

Main Theme 7: Caregiver Engagement and Continuity of Care

Sub-theme 7.1: Continuity Across the Care Pathway

Participants indicated comprehensive cleft care as a continuing pathway rather than a single treatment episode. Their experiences included referral, assessment, nutritional rehabilitation, surgery, follow-up, speech and dental services, psychosocial support, and home-based care activities. The sequence and timing of services varied according to the child’s condition and readiness. Follow-up appointments allowed caregivers to receive additional guidance and continue to the next stages of care. Participants valued being informed about what would happen next and being contacted or reminded about appointments. “After the palate surgery, we continued with dental care and speech therapy. The speech became better, and they also taught us what to do at home. The care continued rather than ending after surgery” (P-9).

Sub-theme 7.2: Caregiver Engagement and Participation

Caregivers reflected that they participated actively in decisions and activities related to their children’s care. Providers explained treatment plans, follow-up requirements, and next steps, allowing caregivers to ask questions and seek clarification. Communication before appointments helped some caregivers prepare for follow-up visits. Participants also described adapting their schedules and household responsibilities to attend appointments. Practical constraints such as illness, transportation, financial limitations, and competing family responsibilities influenced this engagement. “They call us before the appointment, so we know when to come. I ask questions when I do not understand, and they explain what will happen and what we should do next” (P-12).

Main Theme 8: Perceived Changes and Broader Experiences of Care

Sub-theme 8.1: Perceived Functional Changes

Participants reported perceived changes in feeding, physical appearance, speech, and general functioning following different components of care. Some reported that their children had fewer feeding difficulties after nutritional support and surgery and could eat foods that had previously been difficult to manage. Participants also described perceived improvements in speech and communication following speech-related interventions and home-based practice. These findings represent caregivers’ perceptions and experiences and should not be interpreted as objective measures of clinical effectiveness. “After the nutrition support and surgery, feeding became easier. The child could eat solid food, and the appearance also changed. The child started making sounds like ‘baba’” (P-6).

Sub-theme 8.2: Psychosocial and Social Reintegration

Participants indicated perceived changes in their children’s social interaction and participation. Before treatment, some caregivers reported hiding their children, avoiding public places, or feeling anxious about how others would respond. Following treatment, caregivers perceived that their children were more willing to interact with others and participate in everyday social activities. Some caregivers also described reduced personal distress and greater willingness to take their children into the community. These accounts suggest perceived psychosocial and social benefits associated with the care experience but do not constitute objective measures of quality of life or social participation. “Before treatment, I used to hide the child when we traveled by bus because I was afraid of people’s reactions. Now I feel different because the child looks better, and we can go out and interact with others” (P-5).

Sub-theme 8.3: Satisfaction with Comprehensive Care

Participants generally described satisfaction with receiving multiple components of care within one coordinated pathway. They valued combining surgery with nutrition, speech, dental, psychosocial, and follow-up services. Caregivers emphasized that their experience extended beyond the surgical procedure and included continued support and communication. Respectful treatment, clear explanations, emotional support, and access to different services were repeatedly identified as important aspects of a satisfactory care experience. “It is comprehensive because it does not end with surgery. There are speech therapy, dental care, psychosocial support, and follow-up. They care for the child” (P-11).

Sub-theme 8.4: Advocacy and Community Awareness

Some respondents described changes in their own attitudes toward cleft conditions and a willingness to share their experiences with others. Having learned that clefts could be treated, participants reported wanting to challenge myths, encourage families not to hide affected children, and promote early treatment-seeking. Several caregivers expressed a desire to tell other families about available services and the importance of continuing follow-up. Their accounts suggested that personal care experiences could become a source of informal community education and advocacy. “Families should not lose hope or be ashamed. They should seek treatment early and continue the follow-up. I want to tell others about what we experienced so that they also know that treatment is possible” (P-10).

## 4. Discussion

The analysis ultimately identified eight overarching themes that captured caregivers’ lived experiences of accessing comprehensive cleft care at JUMC. Collectively, these themes provide a comprehensive understanding of the challenges, facilitators, perceptions, and experiences caregivers encountered while accessing CCC services in southwestern Ethiopia. An important distinction in interpreting these findings is between access to cleft surgery and access to comprehensive, longitudinal cleft care. Much of the existing literature on cleft care in resource-constrained settings focuses primarily on access to surgical treatment, including barriers related to transportation, geographic distance, referral pathways, treatment costs, and availability of surgical services.

While these barriers were also evident in our participants’ experiences, this study examined a broader continuum of care beyond surgery, including nutritional rehabilitation, speech and language support, dental care, psychosocial support, multidisciplinary assessment, and follow-up. Consequently, access to comprehensive cleft care involved multiple points of engagement with the health system, and barriers were not necessarily uniform across these components.

The experiences described by caregivers therefore suggest that access to comprehensive cleft care is a longitudinal process rather than a single event culminating in surgery. Although surgical treatment was an important component of the care pathway, caregivers’ experiences also reflected the practical and emotional demands of engaging with multiple services over time. This broader perspective helps explain why some barriers persisted even after caregivers had entered the comprehensive cleft care program and why continuity of care remained challenging for some families.

### 4.1. Initial Experiences and Awareness of Cleft Conditions

Caregivers in this study had limited knowledge of orofacial cleft conditions, and community myths and cultural beliefs strongly shaped their experiences. Orofacial cleft conditions were often blamed on supernatural powers, ancestral curses, or divine punishment, resulting in stigma, social isolation, marital conflict, and hiding of affected children. Similar findings from Rwanda and Ghana reported that poor awareness and cultural beliefs delayed treatment-seeking, and some caregivers sought help from traditional healers [22,27].

Most children with orofacial clefts were diagnosed only after childbirth, reflecting gaps in prenatal ultrasound detection by frontline healthcare providers. Participants reported delayed diagnosis and referral as limiting opportunities for early counseling, feeding support, and timely intervention. Studies from several African countries have reported that caregivers are often not informed about their child’s cleft condition during pregnancy because prenatal identification may be missed or information may not be communicated effectively, which can contribute to delays in seeking specialized care [12].

Participants indicated becoming aware of their newborn’s cleft condition as a distressing experience, associated with feelings of loneliness, stress, trauma, shock, despair, and, in some accounts, suicidal ideation. These findings underscore the importance of integrating psychosocial support alongside surgical care [37]. Indeed, this is not as straightforward as it is in high-resource settings, where care is better facilitated and well-established multidisciplinary and continuum-of-care pathways are already in place [14,37].

### 4.2. Caregivers’ Journey to Access Comprehensive Cleft Care

The pathway from recognition of a child’s cleft condition to receiving comprehensive care can be understood using Thaddeus and Maine’s Three Delays framework [38]. Caregivers described delays in seeking care associated with stigma, fear of discrimination, and limited awareness, followed by barriers related to transportation costs and geographic access, particularly for families living in rural areas. The third delay, which concerns delays in receiving appropriate care after reaching a health facility, was not a primary focus of this study.

The first delay was described as limited knowledge, stigma, and cultural myths, consistent with findings from Rwanda and Nigeria, where sociocultural beliefs were reported to contribute to delays in seeking treatment [22,29]. The second delay involved geographic and structural barriers, including transportation costs, poor road infrastructure, lodging expenses, and weak referral systems. Similar delays in accessing surgical care have been documented in other resource-constrained settings, where patient-, transportation-, and health-system-related barriers may delay treatment [39].

Caregivers from urban settings described fewer barriers related to stigma and earlier access to care, particularly where maternal literacy and awareness were higher. The unexpected diagnosis of a newborn’s cleft condition after birth was also described as a source of significant emotional distress for caregivers. Caregivers also described delayed recognition of associated cleft palate as contributing to delays in appropriate feeding support and referral, which they linked to feeding difficulties and concerns about inadequate nutrition.

Participants indicated that some children arrived at comprehensive cleft care centers with substantial nutritional difficulties, which they associated with the need for preoperative nutritional rehabilitation before surgical repair. Evidence from cleft-care settings has similarly highlighted the role of preoperative nutritional interventions in improving surgical eligibility among children with orofacial clefts [24,25]. These findings highlight the potential value of integrating nutritional rehabilitation, psychosocial support, and multidisciplinary services within comprehensive cleft-care programs to address the complex needs of children with cleft conditions and their families.

### 4.3. Experiences of Receiving Comprehensive Cleft Care

The perceived value of comprehensive cleft care extended beyond surgical repair and caregivers’ perceptions of changes in their children’s nutritional status. Caregivers described the program as highly beneficial and perceived benefits for their children’s health, functional abilities, and quality of life. They also described the program as helping protect families from financial hardship associated with accessing cleft care and as providing renewed hope, confidence, and optimism about their children’s future. Other studies have reported similar experiences. For example, the Eurocleft study found associations between treatment outcomes, patient and parent satisfaction, and burden of care, highlighting the broader implications of coordinated cleft care beyond surgical outcomes [40].

Studies from resource-limited settings have documented transportation, financial, geographic, and referral barriers to accessing cleft surgical services [12,22,23]. These barriers also affect comprehensive cleft care, but their implications may differ when care requires repeated engagement with multiple services over an extended period. In the present study, participants described challenges not only in reaching specialized treatment but also in maintaining engagement with nutrition, rehabilitation, psychosocial, and follow-up services.

Thus, evidence describing barriers to surgical access provides important context for understanding our findings but may not fully capture the additional demands of longitudinal, multidisciplinary cleft care. In Sub-Saharan Africa, these barriers may include substantial indirect costs associated with accessing specialized services, particularly for families living far from treatment centers [12,22]. These findings suggest that establishing an integrated cleft care center locally, such as at JUMC, may not by itself ensure equitable access unless financial, geographic, transportation, and referral barriers are also addressed.

### 4.4. Social and Contextual Influences on Access to Care

Myths, stigma, and religious misconceptions surrounded orofacial cleft conditions in the study setting. Participants described beliefs that attributed the condition to ancestral curses or divine punishment affecting the maternal or paternal family. Similar findings have been reported in Ghana and Rwanda, where cultural beliefs and limited awareness were described as contributing to delayed treatment-seeking and psychosocial challenges for caregivers [22,27]. Experiences of stigma, discrimination, and social and structural inequalities have also been reported among families affected by cleft conditions in other low- and middle-income settings [28].

Accessing comprehensive cleft care also imposed significant financial hardship on caregivers. Participants described transportation, accommodation, and daily living expenses as substantial barriers to accessing care, with some families reporting the need to sell assets or discontinue treatment. Similar access barriers have been documented in Rwanda and Madagascar, particularly in relation to geographic and financial constraints affecting access to specialized surgical services [22,23].

Community support was inconsistent, with some neighbors offering financial assistance while others perpetuated stigma. Participants indicated that external support from a non-profit organization helped them overcome financial barriers and access multidisciplinary cleft care. These findings underscore the importance of considering financial and social support mechanisms alongside clinical services when seeking to improve equitable access to comprehensive cleft care.

### 4.5. Experiences Within the Healthcare System

Participants indicated substantial changes in their emotional experiences after accessing comprehensive cleft care at Jimma University Medical Center. Before their children received care, many caregivers reported feelings of fear, shame, isolation, uncertainty, and hopelessness about their children’s futures. After enrolling in the CCC program, caregivers described relief, renewed hope, confidence, and optimism. They viewed the center as a place of dignity, emotional support, and reassurance for the entire family, in addition to its role in providing surgical treatment.

These findings are consistent with qualitative studies from Rwanda and Ghana that have described substantial emotional and psychosocial challenges among caregivers of children with cleft conditions and the importance of comprehensive and supportive care [22,27]. In contrast, evidence from established cleft-care settings has highlighted concerns related to speech, facial appearance, psychosocial well-being, and quality of life alongside clinical treatment outcomes [40,41].

Caregivers highly valued providers’ compassionate, respectful, and non-judgmental approach, which they described as fostering trust and reducing fear of the healthcare system. Caregivers of children with orofacial clefts in other low- and middle-income settings reported similar experiences, describing supportive interactions with healthcare providers as important for coping with the challenges of cleft care [29,30]. These findings point to the importance of compassionate, patient-centered communication within comprehensive cleft care. They also suggest that integrated, multidisciplinary services may address caregiver needs beyond surgical treatment [22,40].

### 4.6. Caregiver Adaptation and Capacity

Key factors influencing caregiver engagement with comprehensive cleft care included caregivers’ knowledge, emotional adaptation, and trust in healthcare providers. Initially, limited knowledge and misconceptions were associated with fear, stigma, emotional distress, and uncertainty. Interviewees explained how CCC interventions, including counseling, health education, nutritional rehabilitation, feeding demonstrations, and peer support, increased their knowledge, confidence, and self-efficacy and supported continued engagement in long-term follow-up care.

Our findings align with studies from South India, Europe, and other low- and middle-income countries that highlight the importance of caregiver education, psychosocial support, family-centered care, interdisciplinary treatment, and caregiver adaptation [30,31,32,33,34,42]. The results indicate the importance of strengthening caregiver education, psychosocial counseling, practical skills development, and trusting relationships between caregivers and healthcare professionals as integral components of comprehensive cleft care, particularly in resource-limited settings where misconceptions and stigma persist.

### 4.7. Caregiver Engagement and Continuity of Care

Caregivers highly valued providers’ compassionate, respectful, and non-judgmental approach, which they described as fostering trust and reducing fear of the healthcare system. Caregivers and healthcare professionals in other low- and middle-income settings have reported similar experiences, highlighting the importance of effective communication, multidisciplinary collaboration, and supportive relationships within cleft-care services [29,30,43]. The longitudinal nature of comprehensive cleft care also meant that access could not be understood solely in terms of whether a child reached surgery.

Participants reported repeated interactions with nutrition, speech, dental, psychosocial, and follow-up services, each of which could introduce additional barriers. Consequently, a family may successfully access surgical treatment while continuing to experience barriers to subsequent rehabilitation or follow-up. This distinction matters because surgical access is one point in a longer continuum of care. Kantar et al. highlighted the importance of financial support and patient-centered service delivery in comprehensive cleft care and identified geographic and demographic factors as important considerations for access to services in low-resource settings [19].

These findings align with those from Ghana and Rwanda, where multidisciplinary coordination, referral pathways, and collaborative healthcare teams were identified as key components of comprehensive cleft care, while financial constraints, geographic distance, and referral delays remained barriers to accessing treatment [22,27]. Evidence from African cleft-care programs and other low-resource settings similarly highlights the importance of integrated multidisciplinary services, patient-centered care, and financial support mechanisms in supporting equitable access and continuity of care [12,19].

### 4.8. Perceived Changes and Broader Experiences of Care

Caregivers indicated perceived benefits of comprehensive cleft care beyond surgical repair, including perceived changes in children’s feeding, nutritional status, speech, appearance, and overall health. Caregivers linked these perceived functional changes to reduced stigma, increased self-confidence, greater social participation, and improved quality of life. These caregiver-reported experiences complement clinical evidence from Ethiopia documenting postoperative outcomes following cleft repair [26]. Caregivers highly valued coordinated multidisciplinary services, respectful provider–caregiver relationships, psychosocial support, and ongoing follow-up, with several becoming advocates for early treatment within their communities.

These findings are consistent with international cleft-care standards that emphasize multidisciplinary management and attention to functional, psychosocial, and patient-centered outcomes alongside surgical care [16]. Similarly, the Eurocleft study reported higher patient and parent satisfaction and a lower burden of care related to treatment outcomes within coordinated cleft-care services [40]. Stock and Feragen highlighted the importance of appearance, communication, social experiences, and psychological adjustment in the lives of individuals with cleft [41]. Similarly, Chung et al. documented the psychosocial impact of stigma among individuals with clefts in low-resource settings [14]. Together, these findings support conceptualizing comprehensive cleft care as a holistic, family-centered model that integrates surgical, rehabilitative, psychosocial, and educational interventions and addresses the broader health and well-being needs of children with cleft conditions and their families.

Caregivers’ accounts revealed a persistent tension between the support they experienced after accessing specialized services and the substantial barriers they faced before and during the care pathway. Delayed recognition and referral, geographic distance, transportation and accommodation costs, stigma, family abandonment, and emotional distress continued to shape caregivers’ experiences, even when clinical and psychosocial services were available. For some families, these challenges contributed to delayed treatment, interrupted care, or substantial emotional and financial burden. At the same time, participants reported compassionate providers, financial and practical support, multidisciplinary services, and treatment-related improvements as important sources of reassurance and hope.

These contrasting accounts suggest that comprehensive cleft care may provide meaningful support without fully resolving the structural and social conditions that shape access to and continuity of care. The lived experience of caregivers was therefore characterized not simply by transformation through treatment, but by navigating between meaningful sources of support and persistent vulnerability. In this context, access to surgery represents an important event within the cleft care pathway, whereas access to comprehensive cleft care represents an ongoing process requiring sustained engagement with multiple services over time.

Overall, caregivers’ experiences indicate that comprehensive cleft care was an important source of clinical, emotional, and practical support, but its availability did not eliminate broader barriers to access and continuity of care. The coexistence of renewed hope and persistent distress, supportive healthcare relationships and inadequate social support, and access to specialized treatment alongside transportation and financial difficulties underscores the complexity of caregivers’ experiences. These findings suggest that strengthening comprehensive cleft care requires not only multidisciplinary clinical services but also attention to timely referral, geographic and financial accessibility, psychosocial needs, stigma, and the social circumstances in which caregivers provide long-term care.

## 5. Limitations

This study was conducted at a single tertiary healthcare center, JUMC, and the findings may not be automatically transferable to other settings in Ethiopia, where the organization and availability of comprehensive cleft care may differ. The purposive sample consisted predominantly of mothers, with limited representation of fathers and other caregivers, and did not include the perspectives of children with orofacial clefts or healthcare providers. Eligibility was limited to caregivers who had used at least two components of the comprehensive cleft care program, which may have excluded families with limited engagement with services. Although participants were reassured about the confidentiality and non-evaluative nature of the interviews, social desirability bias may have influenced their responses. Despite these limitations, the study provides an in-depth account of caregivers’ experiences of comprehensive cleft care at JUMC.

## 6. Conclusions

This study provides an in-depth exploration of caregivers’ experiences of comprehensive cleft care at JUMC. Participants described a care journey that often began with limited awareness of orofacial clefts, emotional distress, sociocultural stigma, financial hardship, and challenges accessing specialized services. Through their engagement with comprehensive cleft care, caregivers reported increased understanding of their child’s condition, enhanced confidence and caregiving skills, greater trust in healthcare providers, and perceived improvements in their children’s health, functioning, and social participation. Many also described reduced experiences of stigma and greater social acceptance over time. Despite these positive experiences, participants highlighted ongoing barriers related to poverty, transportation costs, geographical distance, delayed referral, and limited awareness of available services. These findings provide context-specific insight into caregivers’ experiences of longitudinal multidisciplinary cleft care and highlight important considerations for policy and program planning to strengthen comprehensive cleft care and address barriers to access and continuity of care in similar settings in Ethiopia.

## Figures and Tables

**Table 1:** Socio-demographic characteristics of in-depth interview respondents at Jimma University Medical Center (N = 12 caregivers).

Characteristics of Respondents	Category	Frequency
Residence	Urban	4
	Rural	8
Marital Status	Married	10
	Divorced	1
	Widowed	1
Relationship with a child	Mother	10
	Grandmother	1
	Father	1
Educational Level	Illiterate	4
	Grade 2	2
	Grade 7	1
	Grade 8	2
	Grade 9	1
	Grade 10	1
	Grade 12	1
Employment Status	Farmer	3
	Housewife	6
	Unemployed	1
	Daily worker	1
	Small business (merchant)	1
Type of Cleft Condition	Unilateral Cleft Lip & Palate (UCLP)	8
	Bilateral Cleft Lip & Palate (BCLP)	4
Family History of Cleft	Yes	4
	No	8

**Table 2:** Emergent themes and categories of caregivers’ lived experiences in accessing comprehensive cleft care at Jimma University Medical Center in Southwest Ethiopia, 2026.

Themes	Categories (Sub-Themes)
Initial experiences and awareness of cleft conditions	Prior knowledge and community perception Diagnosis experience Emotional reaction
Caregivers’ journey to access comprehensive cleft care	Treatment-seeking journey and delay Key events facilitating or hindering access
Experience of receiving comprehensive cleft care	Care dynamics and perceived value of comprehensive cleft care
Social and contextual influences on access to care	Cultural perception and stigma Family and social support networks Healthcare access and logistical barriers Institutional and external support
Experiences within the healthcare system	Patient–provider relationship and trust Interdisciplinary care coordination
Caregiver adaptation and capacity	Change in knowledge and understanding Emotional adaptation Caregiver competence and confidence
Caregiver engagement and continuity of care	Continuity across the care pathway Caregiver engagement and participation
Perceived changes and broader experiences of care	Perceived functional change Psychosocial and social reintegration Satisfaction with comprehensive care Advocacy and community awareness

## Data Availability

The datasets generated and analyzed during the current study are available from the corresponding author upon reasonable request. To protect participant confidentiality, qualitative interview transcripts and other potentially identifiable data are not publicly available.

## Supplementary Materials

The following supporting information can be downloaded at https://www.mdpi.com/article/10.3390/children13101372/s1, File S1: COREQ checklist, Consolidated criteria for reporting qualitative studies (COREQ): 32-item checklist [44].

## Abbreviations

CCC	Comprehensive cleft care
JUMC	Jimma University Medical Center
ORFs	Orofacial clefts
LMICs	Low- and middle-income countries
