# Supplementary material for: Exploring Caregivers Lived Experiences in Accessing Comprehensive Cleft Care at Jimma University Medical Center: A Phenomenological Study in Southwest Ethiopia
Source: Children (Basel). 2026 Oct 10;13(10):1372. doi: 10.3390/children13101372 (PMC13703069; doi:10.3390/children13101372)
Supplement: Supplementary file 1 [file children-13-01372-s001.zip › children-4573928-supplementary.pdf]

Supplementary File S1: COREQ checklist
Consolidated criteria for reporting qualitative studies (COREQ): 32-item checklist

Developed from: Tong A, Sainsbury P, Craig J. Consolidated criteria for reporting qualitative research (COREQ): a 32-item checklist for interviews and focus groups. International Journal for Quality in Health Care. 2007. Volume 19, Number 6: pp. 349 – 357

Item No	Guide Questions/Description	Reported part/explanation
**Domain 1: Research team and reflexivity**		
**Personal Characteristics**		
**1. Interviewer/ facilitator**	**Which author/s conducted the interview or focus group? experience**	**Corresponding author Abiy Semunigus conducted the interviews at Jimma University Medical Center. The Medical Center provided a quiet and private room for the interviews. A trained qualitative researcher fluent in both Afaan Oromoo and Amharic language supported the interview process to facilitate effective communication with participants.**
**2. Credentials**	**What were the researcher's credentials? E.g., PhD, MD**	**The corresponding and first author, Abiy Semunigus, is a final-year PhD candidate in the Department of Health, Behavior, and Society, Faculty of Public Health, Institute of Health, Jimma University. He holds a Master of Public Health (MPH) and a Master of Science (MSc) degree. The remaining four co-authors hold PhD degrees and contributed to the study in accordance with their respective roles.**

Item No	Guide Questions/Description	Reported part/explanation
3. Occupation	What was their occupation at the time of the study?	Abiy Semunigus is a final-year PhD candidate. Dr. Allyn Auslander is AVP of research at Operation Smile, and Dr. Elias , Dr. Fira, and Dr.Abraham are professors at Jimma University.
4. Gender	Was the researcher male or female?	The primary researcher is Male
5. Experience and training	What experience or training did the researcher have?	Abiy Semunigus is a final-year PhD candidate in health communication and behavior. He has PhD-level training in qualitative study
Relationship with participants		
6. Relationship established	Was a relationship established prior to study commencement?	The primary researcher established rapport with participants before each interview to foster trust and create a comfortable, respectful, and non-judgmental environment. This conducive environment encouraged participants to openly discuss their experiences and perceptions.
7. Participant knowledge of the interviewer	What did the participants know about the researcher? e.g. personal goals, reasons for doing the research?	Before the interviews, participants were informed about the researcher's academic role, the purpose of the study, and the interview objectives. Participants were also informed that their participation was voluntary and that their responses would be treated confidentially and would not affect their access to care or services

Item No	Guide Questions/Description	Reported part/explanation
8. Interviewer characteristics	What characteristics were reported about the interviewer/facilitator? e.g. Bias, assumptions, reasons and interests in the research topic	The primary interviewer, Abiy Semunigus, was a final-year PhD candidate in the Department of Health, Behavior, and Society, Jimma University, with postgraduate training in public health and professional experience in comprehensive cleft care. The researcher was aware of the potential influence of his professional background on the interview process and maintained a neutral and non-judgmental approach during data collection. The researcher established rapport with participants before each interview to facilitate open discussion of their experiences and perceptions.
Domain 2: study design		
Theoretical framework		
9. Methodological orientation and Theory	What methodological orientation was stated to underpin the study? e.g. grounded theory, discourse analysis, ethnography, phenomenology, content analysis	This is a phenomenological descriptive study design.
Participant selection		
10. Sampling	How were participants selected? e.g., purposive, convenience, consecutive, snowball	Participants were purposively selected from caregivers who had attended at least two components of comprehensive cleft care services at Jimma

Item No	Guide Questions/Description	Reported part/explanation
		University Medical Center. This criterion ensured participants had sufficient experience with comprehensive cleft care to provide detailed accounts of their experiences and perceptions.
11. Method of approach	How were participants approached? e.g., face-to-face, telephone, mail, email	It was face-to-face
12. Sample size	How many participants were in the study?	Twelve participants
13. Non-participation Setting	How many people refused to participate or dropped out? Reasons?	Noon
14. Setting of data collection	Where was the data collected? e.g., home, clinic, workplace	Data were collected at Jimma University Medical Center in Jimma, Ethiopia. The interviews were conducted in a quiet and private room provided by the Medical Center to ensure confidentiality and minimize interruptions. Participants were interviewed individually in this setting.
15. Presence of nonparticipants	Was anyone else present besides the participants and researchers?	No
16. Description of sample	What are the important characteristics of the sample? e.g. demographic data, date	A total of 12 caregivers participated in the study. Of these, 10 (83.3%) were mothers, one (8.3%) was a father, and one (8.3%) was a grandmother. Eight participants (66.7%) resided in rural areas and four (33.3%) in urban areas. Most participants were

Item No	Guide Questions/Description	Reported part/explanation
		married (10, 83.3%), while one (8.3%) was divorced and one (8.3%) was widowed. Educational attainment varied, with four participants (33.3%) reporting no formal education and the remainder having completed between Grade 2 and Grade 12. Regarding employment, six participants (50.0%) were housewives, three (25.0%) were farmers, and one each (8.3%) was unemployed, a daily worker, or a small-business owner.
	**Data collection**	
17. Interview guide	Were questions, prompts, and guides provided by the authors? Was it pilot tested?	A semi-structured interview guide was developed by the research team based on the study objectives and the experiences of caregivers receiving comprehensive cleft care. The guide included open-ended questions and follow-up prompts to facilitate in-depth exploration of participants' experiences and perceptions. The interview guide was pilot tested with caregivers who met the study eligibility criteria but were not included in the final sample. Based on the pilot interviews, the wording and sequence of questions were refined to improve clarity and flow.
18. Repeat interviews	Were repeat interviews carried out? If yes, how many?	NO

Item No	Guide Questions/Description	Reported part/explanation
**19. Audio/visual recording**	**Did the research use audio or visual recording to collect the data?**	**Yes**
**20. Field notes**	**Were field notes made during and/or after the interview or focus group?**	**Yes**
**21. Duration**	**What was the duration of the interviews or focus group?**	**40-90 minutes**
**22. Data saturation**	**Was data saturation discussed?**	**Yes**
**23. Transcripts returned**	**Were transcripts returned to participants for comment and/or correction?**	**Following the seventh step of Colaizzi's method, the primary researcher presented a summary of the findings to three randomly selected participants from the 12 participants for member checking**
**Domain 3: analysis and findings**		
**Data analysis**		
**24. Number of data coders**	**How many data coders coded the data?**	The primary researcher conducted the main coding and theme development, while a second qualitative researcher independently reviewed two transcripts and provided feedback on the coding and interpretation.
**25. Description of the coding tree**	**Did the authors provide a description of the coding tree?**	**Data were analyzed inductively using Colaizzi's descriptive phenomenological method. The authors identified significant statements, formulated meanings, grouped them into categories, and synthesized themes representing participants' lived experiences.**

Item No	Guide Questions/Description	Reported part/explanation
26. Derivation of themes	Were themes identified in advance or derived from the data?	Derived from data
27. Software	What software, if applicable, was used to manage the data?	ATLAS ti version 7.5.18
28. Participant checking	Did participants provide feedback on the findings?	A summary of the findings was presented to three participants (25% of the sample), selected at random, to confirm whether the identified themes accurately reflected their lived experiences. Participants provided feedback, and the authors incorporated minor clarifications into the final analysis.
Reporting		
29. Quotations presented	Were participant quotations presented to illustrate the themes/findings? Was each quotation identified? e.g., participant number	Yes. Direct quotations from participants were presented to illustrate and support the identified themes and findings. Each quotation was identified using a unique participant number (e.g., P-1, P-2 , P-3 etc) to maintain confidentiality while demonstrating the connection between the findings and participants' accounts.
30. Data and findings consistent	Was there consistency between the data presented and the findings?	Yes
31. Clarity of major themes	Were major themes clearly presented in the findings?	The findings section clearly presented eight major themes and 21 subthemes, using illustrative participant quotations to support each theme.

Item No	Guide Questions/Description	Reported part/explanation
**32. Clarity of minor themes**	**Is there a description of diverse cases or a discussion of minor themes?**	**Yes. The analysis considered and discussed contrasting experiences and divergent perspectives within the relevant themes and subthemes. The analysis did not focus solely on positive experiences; both positive and challenging experiences were incorporated to provide a balanced representation of caregivers lived experiences.**
